# Progesterone Treatment Shows Benefit in Female Rats in a Pediatric Model of Controlled Cortical Impact Injury

**DOI:** 10.1371/journal.pone.0146419

**Published:** 2016-01-22

**Authors:** Rastafa I. Geddes, Bethany L. Peterson, Donald G. Stein, Iqbal Sayeed

**Affiliations:** Department of Emergency Medicine, Emory University, Atlanta, GA 30322 United States of America; Brock University, CANADA

## Abstract

**Purpose:**

We recently showed that progesterone treatment can reduce lesion size and behavioral deficits after moderate-to-severe bilateral injury to the medial prefrontal cortex in immature male rats. Whether there are important sex differences in response to injury and progesterone treatment in very young subjects has not been given sufficient attention. Here we investigated progesterone’s effects in the same model of brain injury but with pre-pubescent females.

**Methods:**

Twenty-eight-day-old female Sprague-Dawley rats received sham (n = 14) or controlled cortical impact (CCI) (n = 21) injury, were given progesterone (8 mg/kg body weight) or vehicle injections on post-injury days (PID) 1–7, and underwent behavioral testing from PID 9–27. Brains were evaluated for lesion size at PID 28.

**Results:**

Lesion size in vehicle-treated female rats with CCI injury was smaller than that previously reported for similarly treated age-matched male rats. Treatment with progesterone reduced the effect of CCI on extent of damage and behavioral deficits.

**Conclusion:**

Pre-pubescent female rats with midline CCI injury to the frontal cortex have reduced morphological and functional deficits following progesterone treatment. While gender differences in susceptibility to this injury were observed, progesterone treatment produced beneficial effects in young rats of both sexes following CCI.

## Introduction

Traumatic Brain Injury (TBI) has a world-wide incidence rate of 106 per 100,000 population [1], and no FDA-approved therapy currently exists [2,3]. Globally, adolescents have the highest TBI rates of any age group [4–7] and males are nearly three times as likely as females to die from a TBI [1]. Current statistics suggest that gender may play a role, with females lower in TBI susceptibility, extent of injury and prognosis.

Although progesterone (PROG) has been shown to be beneficial in pre-clinical laboratory research in multiple models of central nervous system (CNS) injuries including TBI [8–15], several dozen trials over the last 15–20 years attempting to treat adult TBI have all produced negative outcomes [16], and two recent Phase III clinical trials, SyNAPSe (ClinicalTrials.gov Identifier: NCT01143064) and ProTECT III (NCT01143064), reported no significant beneficial effects of acute PROG treatment on moderate to severe closed-head TBI in adult males and females [17,18]. Unfortunately, these trials did not conduct dosing and duration of treatment optimization studies prior to testing the hormone in patients and had other design problems [19,20]. In addition, although the trials did not directly study sex differences, sex differences in variability of injury severity, outcomes, dose-optimization [19], post-acute rehabilitation, and co-morbidities could have been a factor in the results (see [20,21] for more details).

Whether PROG’s neuroprotective effects after brain injury vary in males and females across the developmental spectrum is still an open question. For example, following neonatal hypoxic-ischemic injury in both male and female rats, PROG-treated males surprisingly showed much more substantial tissue sparing and less reactive gliosis than females and there were significant sex differences in behavioral outcomes when the animals were tested later in life [22]. However, in c57BALB mice, sex differences in response to a cortical contusion injury were seen in only a few measures of activity—in cognitive and motor tasks, the deficits were the same [23]. Recent individual animal meta-analyses of a number of published preclinical studies of PROG in females with stroke [24] showed an increase in the incidence of stroke-related death in adult females, highlighting the need for investigations to evaluate how the female subject may differentially respond to brain injury.

PROG is an important sex steroid as well as a developmental hormone, so young females with brain injury just entering puberty/estrus may be more susceptible to rapid changes in hormonal levels of PROG that could result in different morphological and functional outcomes compared to male conspecifics or older subjects with similar damage. Robertson et al. [25] reported that tissue loss was reduced in PROG-treated female rats at 7d after unilateral contusion injury to the exposed brain and suggested a need for future studies looking at functional outcome measures. Recently, we showed that PROG treatment reduced lesion size and behavioral deficits after moderate-to-severe bilateral injury to the medial prefrontal cortex (mPFC) in post-natal day (PND) 28 male Sprague-Dawley (SD) rats [26]. Here we report on the response of pre-pubescent female rats with a similar TBI to post-injury PROG treatment. We think it is important to analyze responses to brain injury by gender as well as by stage of development, especially when critical sex hormones may affect functional and morphological outcomes. We tested the hypothesis that PND 28 female rats with a controlled cortical impact (CCI) injury would show the same benefits of neurosteroid treatment as their age-matched male conspecifics. Rats do not become sexually mature until about 6–8 weeks [27], so the present study reports a model of pediatric brain injury for evaluating PROG treatment following TBI in animals at an age range equivalent to that of a 9-10-year-old human child. This study could provide important information in designing future clinical trials of PROG treatment in children and adolescents with TBI.

## Materials and Methods

### Subjects

Forty-eight SD rats (Harlan) were acquired at PND 21 and acclimated to the environment over 2 days. Rats were weighed on PND 23–25 and daily thereafter and housed, fed and maintained on a 12-hour reverse light/dark cycle. The Institutional Animal Care and Use Committee of Emory University approved the procedures used in this study and the research was conducted in an AAALAC-approved facility (Protocol # 2001801).

### Surgery

Surgeries were performed on PND 28. For initial anesthesia, the rats were placed in an air-tight induction chamber with oxygen, nitrous oxide, and isoflurane gas (4% induction, 1.5% maintenance, 700 mmHg/min N_2_O, and 500 mmHg/min O_2_). They were then mounted in a Kopf stereotaxic device (model 900) equipped with a Mouse and Neonatal Rat Adaptor (model BJK-030). The animal’s head was held in place by non-traumatic ear bars and a bite bar. Anesthesia was delivered just prior to the surgery by nose cone and the rats’ heads shaved and sterilized with 70% ethanol and Betadine^™^ antiseptic solution. Anesthesia levels were monitored closely throughout surgery and were frequently adjusted between 400–700 mmHg/min, based on heart rate and oxygen saturation. In our previous study [26] we found that a 4.0-mm diameter stainless steel impactor resulted in a survivable, severe CCI injury in 28-day-old male rats so we used the same CCI parameters for the age-matched female rats.

A SurgiVet^™^ pulse oximeter (model V3304) was attached to the animal’s rear paw to monitor and maintain blood SpO_2_ at or above 90%. A heart rate monitor with its sensor attached to the other hind paw was used to maintain a rate ≥ 300 beats per minute. A homeothermic blanket control unit (Harvard Apparatus, Holliston, MA) was used to monitor and maintain core body temperature (~37°C) and prevent hypothermia throughout surgery. Under aseptic conditions, the cranium and its bony landmarks including bregma (β) and lambda (λ) were exposed by making a midline incision along the scalp into the skin and fascia covering the skull. The craniectomy was centered on the midline at 2.0 mm anterior to β. The cortical impact was made over the midline medial frontal cortex with an Impact One^™^ Stereotaxic Impactor for CCI (Leica #39463920). The sham-injured groups received the same surgical procedures up to and including craniectomy but no CCI injury. After surgery, the rats were placed on a heating pad, monitored closely and upon awakening were returned to their home cages. Of the 48 females at the beginning of the experiment, 3 died under surgery.

### PROG solution and injection schedule

The remaining 45 rats were randomly assigned to one of 4 groups: CCI+PROG (8 mg/kg; n = 12); CCI+vehicle (n = 11); sham-vehicle (n = 11); and sham+PROG. CCI+PROG received injections of 8 mg/kg of PROG (4-pregnene-3, 20-dione, Sigma Aldrich, St. Louis, MO) dissolved in 22.5% 2-hydroxyropyl-β-cyclodextrin (HBC) (Sigma). The incoming animals were allocated to each of the groups by non-systematic selection and assignment soon after they were delivered to the animal housing quarters. Once assigned to a group they were numbered and then the group was coded for blinding purposes until the completion of the experiment. The solutions containing PROG and vehicle for CCI and sham groups were independently coded prior to administration so that investigators were blinded to the agents being injected.

The dose used here was the same as that found to be most beneficial in our previous experiment in similarly aged males [26]. PROG was administered at a volume of 0.02 ml/100 gm body weight. However, the sham+PROG group was given 16 mg/kg doses of the drug (1) to permit comparisons with the highest dose previously tested in the sham-injured juvenile male rats [26], and (2) to demonstrate the safety of a “higher-than-effective” dose of PROG in our normal or non-injured juvenile female subjects. A total of 9 injections were administered to each animal in all experimental conditions at the following post-injury times: 1, 3, and 24 h, and 2, 3, 4, 5, 6, and 7 days. All injections were given subcutaneously except the first, which was administered intraperitoneally to ensure more rapid absorption following injury [28,29]. To avoid withdrawal effects, the 8^th^ and 9^th^ injections of PROG were tapered (one-half and one-quarter of the original dose, respectively) [30,31]. PROG was prepared just prior to surgery and again on the 4^th^ post-injury night.

### Behavioral testing

We used the same regimen of behavioral testing for the females as for the males [26]. To obtain baseline data, rotarod performance and behavior in the elevated plus maze (EPM) were assessed in all rats prior to surgery. All post-surgery testing was delayed for one week, during which the rats received daily vehicle or PROG injections as described above. Animal IDs were coded to keep experimenters blind to group identity throughout behavioral testing and histological analysis.

#### Anxiety-like behavior in the EPM

In the EPM, anxiety is computed by determining the amount of time spent in the open vs. the closed arms. This is because in novel situations, rats tend to exhibit thigmotactic behavior—huddling next to walls or enclosed spaces, which provide mechanical stimulation. Because lab rats are naturally exploratory, reduced thigmotaxis (inferred from time spent exploring the open arm) is taken as an indication of lowered stress or anxiety. Thus the total number of open vs. closed arm entries was used as the operational dependent measure. If the animal fell off the open arm it was returned to the start position in the center square and the fall recorded. Individual trial data for each rat was used to determine group averages.

Testing was conducted under red light in a quiet environment. Baseline EPM data was obtained between PND 25 and 27 and rats were tested twice post-surgery (on post-injury (PID) 9 and 17). Each trial lasted 5 min, and the total number of open arm entries (or crosses over the center square with both front and hind paws) was reported as percent of visits to the open and closed arms.

#### Rotarod testing

An accelerating Exconomex^™^ rotarod (Columbus Instruments, Columbus, OH) was used under red light to assess balance and motor coordination. Rats were given initial habituation training and then baseline testing prior to surgery. For habituation training, rats were placed on a stationary rod for at least 3 min, then slowly habituated to the rotating rod from a starting speed of 1 rpm, which was accelerated to 30 rpm over 5 min. A baseline score was obtained on PND 27 and rats were again tested on PID 9, 15, 21, 25 and 27. Rats were scored on latency to fall off the rod (maximum score was 300 s), and each testing day consisted of three trials separated by a 10-min break. Scores from the three trials for each day were averaged.

#### Spatial navigation performance and memory in the MWM

MWM tests were conducted under dim white light in a white plastic pool measuring 135 cm in diameter [26]. The rat’s position in the maze, swim distance, and latency to reach the platform was recorded with an overhead camera and computer-assisted tracking system (CleverSys, Reston, VA).

Beginning on PID 10, all the rats were tested for acquisition in the MWM. Each animal received two trials per day, separated by a 5-min interval. A trial consisted of placing the subject in the pool facing the wall and allowing it to swim until it reached the platform or until 90 sec had elapsed. When a rat was unable to locate the MWM platform within the allotted time, the experimenter led the rat to the platform. Rats were allowed to remain on the platform for 20 sec and then removed from the pool. After 5 min, subjects were again released into the tank, but this time from the opposite position from that in the previous trial and allowed to swim to the platform. Animals were placed in holding cages in front of an air heater between trials and before being returned to their home cages.

### Histology

Like the previously tested males, at 1 month post-injury, the females were fatally anesthetized and their brains perfused with saline, then with 10% formalin, and then extracted for histological analysis. The brains were cut into 20-μm sections on a cryostat and stored at −80°C on 1% gelatin coated (subbed) slides. Slides were stained in 0.1% cresyl violet solution (0.1 g cresyl violet acetate and 0.3 ml of glacial acetic acid dissolved in 100 ml dH_2_O) for 10 min at 45°C and then rinsed in distilled water. The percent of damaged issue was identified in six 20-micron Nissl-stained sections and analyzed from between 4.5 and -0.5 mm from β for lesion size using the Image J^™^ System (Media Cybernetics, Silver Spring, MD).

Stained slides were scanned with Silverfast Pathscan software (PathScan Enabler IV, Meyer Instruments, Houston TX) and the scanned images were analyzed using ImageJ^™^. The percentage of injured tissue from a single section was calculated by tracing the perimeter of the injury and determining its surface area, dividing this by an estimate of the total surface area of the section (taken by tracing both the remaining tissue and the estimated perimeter of the necrotic cavity), and multiplying by 100.

### Qualitative observations on females surviving the TBI

**Fig 1:**
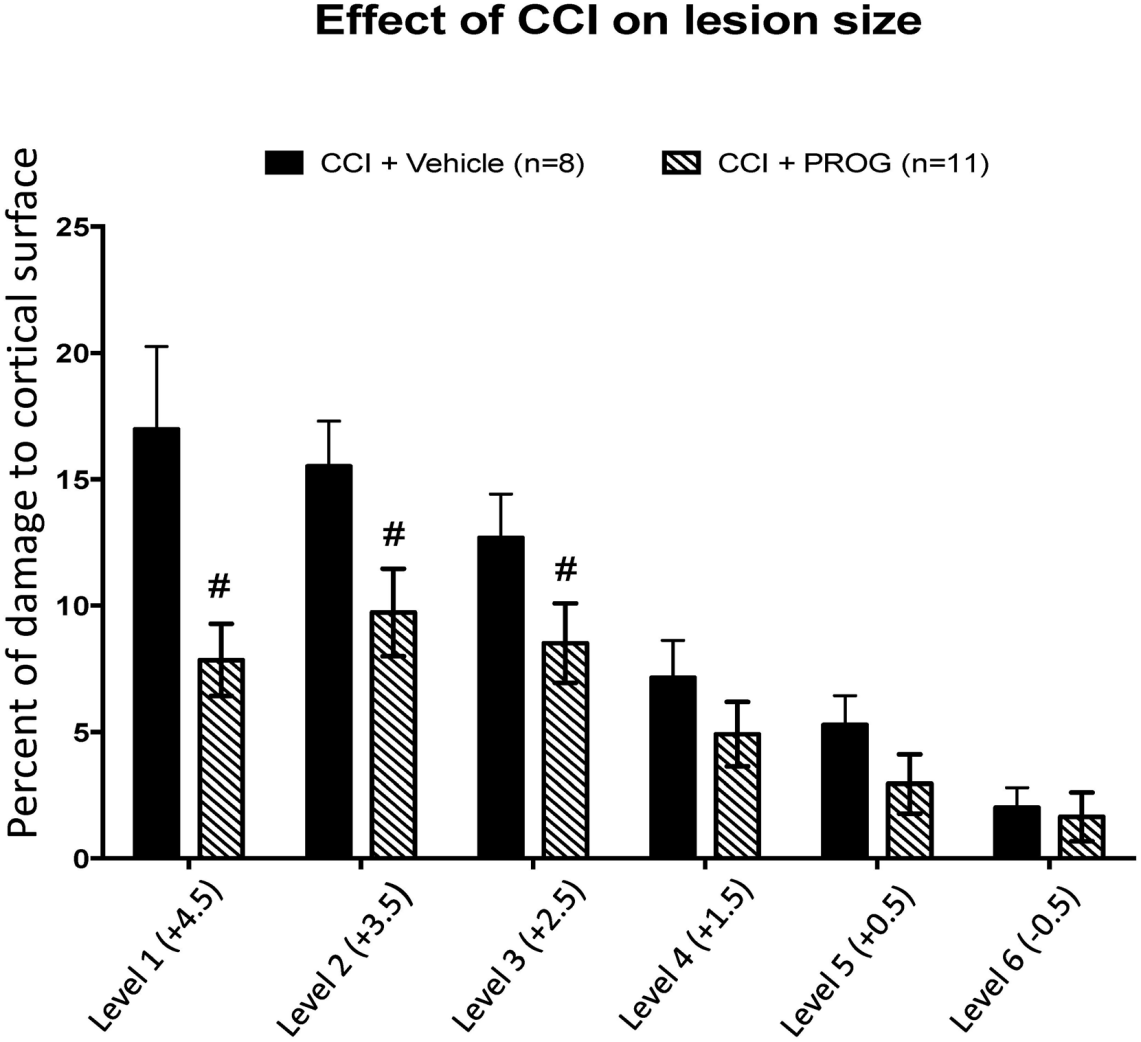
Lesion reconstruction analysis of juvenile female rats with controlled cortical impact (CCI) treated with progesterone (PROG) vs. vehicle. Mean percent (± SEM) of volumetric tissue loss at 6 anterior-to-posterior (A/P) levels at 4 weeks post-injury. # = difference between the CCI+vehicle-treated group and CCI rats given 8 mg/kg PROG. Values are mean ± SEM (n = 6–11 / group).

**Fig 2:**
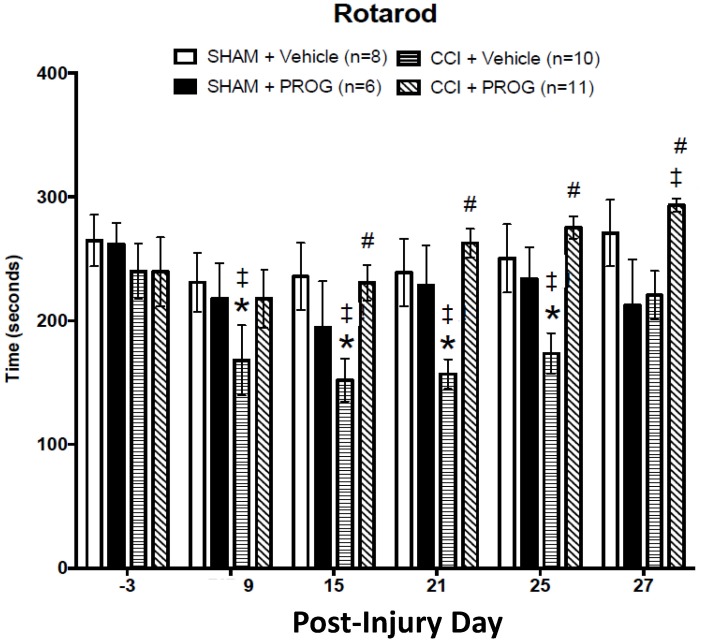
Effect of progesterone (PROG) treatment on vestibulomotor function in female CCI rats treated with PROG vs. vehicle. PROG (8 mg/kg) -treated CCI rats showed improvement in balancing and walking on the rotarod tasks. The Sham+PROG group was given 16-mg/kg doses of PROG. * = different from sham + vehicle; # = different from CCI + vehicle (*p*’s < 0.05). ‡ = different from baseline within each group. Values are mean ± SEM (n = 6–11).

**Fig 3:**
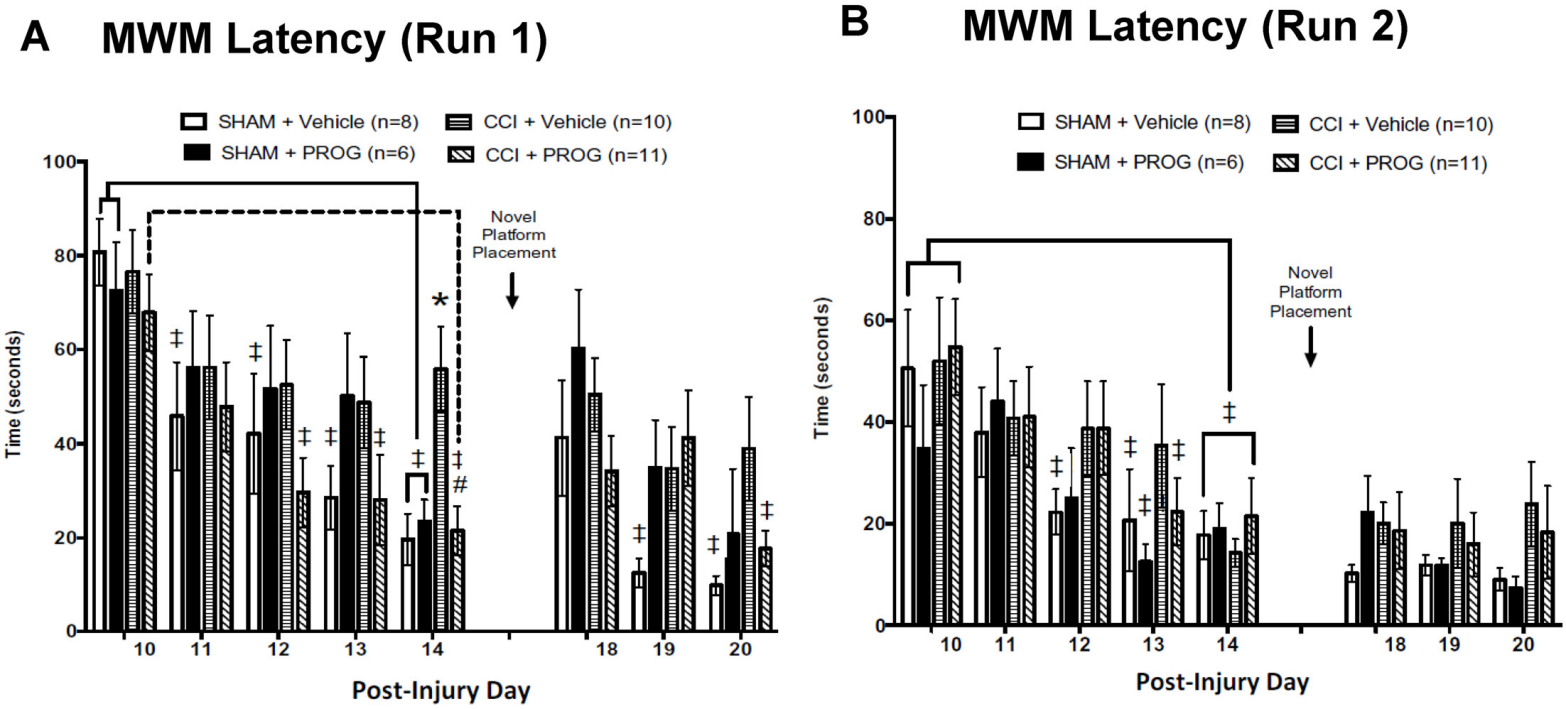
Effects of progesterone (PROG) on learning and memory task as assessed by latency to locate the Morris water maze (MWM) platform. During Run 1 **(a)** on the acquisition phase there was a significant effect of controlled cortical impact injury. Eight mg/kg PROG proved beneficial by decreasing the mean latency to find a hidden platform in the MWM compared to the CCI+vehicle-treated group. There was no clear effect of PROG treatment on Run 2 **(b)** or during the reversal phase (*p* > 0.05). The Sham+PROG group was given 16 mg/kg doses of PROG. * = different from Sham+Vehicle; # = different from CCI+Vehicle (*p*’s < 0.05). ‡ = different from baseline within each group; n = 6–11.

Of the 22 sham rats, the data for 8 were removed from final analysis. The reasons for removal included: failure to meet the criterion of 180 ms on the rotarod during baseline testing (n = 2); evidence of unreliable data during post-surgery testing (n = 5) due to attempts to escape the apparatus or hugging the rod (thus missing the sensor); and an unintentional injury to brain from the drill bit during craniectomy (n = 1). The data for 14 out of 22 sham-injured females (8 in the sham+vehicle and 6 in the sham+PROG groups) are presented in Figs 1–3. Of the 23 surviving rats with CCI injury, one failed to meet rotarod baseline criteria and another repeatedly attempted to escape the apparatus rather than run on the wheel. Thus, the data for 21 out of 23 rats with CCI injury are also presented in Figs 1–3, with n = 10 rats in the CCI+vehicle and n = 11 rats in the CCI+PROG group.

### Statistical Analysis

A mixed factorial analysis of variance (ANOVA) for repeated measures was performed on the behavioral results and lesion reconstruction data, which were expressed as the mean ± SEM. Mean comparisons were used for *post-hoc* analyses of repeated ANOVAs. Independent paired *t*-tests were also used to compare the differences between baseline (pre-injury) and post-injury data when data were normally distributed. Statistical significance was established at *p* ≤ 0.05. Based on a delta-value of 1.5 (using latency to find the hidden platform on the MWM task), we calculated the sample sizes and power needed to reject the null hypothesis (of no differences between the CCI+PROG and CCI+vehicle-treated rats) to achieve >80% power to detect a 30% difference (medium effect). The number of rats per group at these criteria was determined to be 9.

## Results

### Lesion (tissue) reconstruction analysis

Briefly, the 6 sections from each rat brain were photographed with an Epson scanner (and ImagePro^™^ software). Group means of percent of damaged tissue averaged across 6 anterior-to-posterior (A/P) sections were used to determine overall lesion size. One of the sham rats had visible drill-induced injury (≥ 1%) that was unintentional and thus its data was excluded from behavioral statistical analysis. Tissue sections from the remaining sham rats had no observable damage. Results from the lesion reconstruction analysis of CCI-injured animals treated with vehicle or PROG are shown in Fig 1. Compared to intact sham-operated animals, there was a significant lesion effect (F(3,29) = 27.54, *p* < 0.01). *Post-hoc* comparisons further revealed significant differences at A/P brain levels 1 to 3 between PROG and vehicle-treated CCI rats (# = *p* < 0 .05).

### Physiological and functional effects of PND 28 CCI-induced injury

#### Body Weight

One-way ANOVA with repeated measures on PND or PID conducted on the mean pre- and post-injury body weights found no main effect on mean body weight (F(3,31) = 0.1351, *p* > 0.05) (S1 Fig). As might be expected, there was, however, a main effect of PID on mean body weight (F(30,930) = 2265.0, *p* < .001). *Post-hoc* pair-wise comparisons indicated that the group mean body weights were significantly increased over time (*p* < 0.01), except for the first two days post-surgery (*p* > 0.05).

#### Elevated Plus Maze

Repeated measures ANOVA were performed on EPM behaviors. The independent measure was Lesion/Treatment and the dependent measures were the percent of entries in the open vs. closed arms. The ANOVA revealed no significant main effect of Lesion/Treatment on the percent of entries in each arm or total crossings (F’s < 1, *p* > 0.05; see S2 Fig). One-way ANOVA revealed that on all 3 test days, all groups preferred the closed arms of the maze (9 to 1).

#### Rotarod

Activity on the rotarod was measured as latency to fall off the rotating/accelerating rod over a 300-sec period. A repeated 4 x 6 (Lesion/Treatment x Test Days) measures ANOVA, with repeated measure on PID, revealed a main effect of Lesion/Treatment on rotarod performance (F(5,45) = 3.87, *p* < 0.05). *Post-hoc* analysis further revealed that rotarod performance during baseline testing was not different among the groups (*p* > 0.05). As indicated in Fig 2, CCI injury and administration of 8 mg/kg of PROG had a significant effect on rotarod performance in the developing female rats. The rats given a CCI performed significantly worse on PID 9, 15, 21 and 25 (^‡^ = *p* < 0.05) compared to their baseline performance. Rotarod performance in the CCI+PROG group, in contrast, did not differ from baseline on these days and was significantly improved by PID 27 (*p* < 0.05). As also indicated in Fig 2, rotarod performance in the CCI+vehicle group was worse than in the sham+vehicle-treated rats on PID 9, 15, 21 and 25 *p* < 0 .05). The CCI+PROG group was not different from sham+vehicle and had significantly better rotarod scores than CCI+vehicle on PID 15, 21, 25 and 27 (*p* < 0.05). Finally, while the 8 mg/kg dose of PROG reduced performance deficits in the CCI-injured rats, the 16 mg/kg dose noticeably increased the variability in performance in the sham group.

#### Spatial navigation and cognition in the MWM

Mean latency to reach the MWM platform within 90 sec during acquisition and novel platform placement learning served as the dependent measures. Group latency scores from Runs 1 and 2 are shown in Fig 3a and 3b and were analyzed using a 4 x 2 x 9 (Lesion/Treatment x Run x Test Day) mixed factorial ANOVA, with repeated measures on PID.

As discussed below, on the first MWM trial the female rat pups were slow compared to their male counterparts. Among the females, there was a significant main effect of Lesion/Treatment (F(3,31) = 5.94, *p* < 0.01) and Test Day (F(7,217) = 11.82, *p* < 0.01) in finding the submerged MWM platform. As shown in Fig 3a, during Run 1 on the second and third days of acquisition training, the vehicle-treated and CCI+PROG-treated groups did significantly better than on the first trial. On the last day of acquisition training (PID 14), before the platform was moved to a novel location, all rats except those in the CCI+vehicle-treated group performed better than they did on PID 10 (^‡^ = *p* < 0 .05). On PID 14 the latency to reach the platform during Run 1 was significantly delayed in the CCI+vehicle group compared to the CCI+PROG (# = *p* < 0 .05) or sham groups (* = *p* < 0 .05).

*Post-hoc* Tukey’s multiple comparison tests revealed (1) a significant difference between CCI and sham rats treated with vehicle (*p* < 0.05) and (2) a significant difference between PROG- and vehicle-treated CCI rats (*p* < 0.05), but (3) the differences between the CCI+PROG and the sham groups were not significant (*p’s* > 0.05). Finally, as shown in Fig 3a, placing the MWM platform in a novel quadrant was transiently disruptive to the performance pattern observed in sham-injured rats at the end of acquisition (PID 14).

As shown in Fig 3b, neither sham-injured nor CCI female rats appeared to use the Run 1 experience to find the MWM platform more efficiently in Run 2 (which was conducted 5 min after Run 1). Overall, CCI had no effect on acquisition learning and performance on the three novel platform placement trials. Taken together, our data show that the CCI injury was sufficient to produce observable, histopathological, specific behavioral and cognitive deficits in female PND 28 rat pups.

## Discussion

### Sex differences in 1-month-old rat pups with CCI injury

Despite the physiological and cognitive differences between healthy young males and females, there is no consensus on whether human females with a TBI have better functional outcomes than males [32–36]. It is also not clear whether the levels of female hormones like estrogen (or estradiol) and PROG, which increases exponentially during pregnancy, play a role in sex-specific neuroprotection or neurodegeneration following a TBI [37–45]. Interestingly, Wagner et al. [46] found no sex differences in the acute elevation of serum sex hormone levels in adult patients with a severe TBI but noted that elevated levels of testosterone in women and estradiol in men were associated with a poor prognosis and increased mortality at 6 and 12 months after injury. Less is known about whether such outcomes would be observed in younger subjects, so gathering similar data in juveniles could be important in planning for clinical investigation of neurosteroid treatment for male and female juvenile TBI patients [47]. This is not a trivial issue. Two independent studies collectively analyzing 56,994 juvenile patients with moderate-to-severe brain trauma found that adolescent, but not prepubescent, brain-injured girls had a lower incidence of mortality than age-matched brain-injured boys [48,49]. These data were interpreted to suggest that an “active” (and developing) reproductive system affords juvenile females a sex-specific advantage during a TBI event and recovery.

A growing number of preclinical studies show that the effectiveness of post-injury PROG treatment for brain trauma in the developing rat may be affected by age, gender and injury model [50–55]. Mannix et al. [56] recently found that PROG treatment improved grip strength in CCI-injured 8-week-old male mice but worsened performance in females, while the MWM spatial learning deficit was unaffected by PROG treatment in either sex. As noted in our introduction, two recently reported failures in clinical trials of PROG for the treatment of TBI in adults found no benefits of PROG treatment on very blunt and simplified quality of life outcome measures, but potential sex differences in biomarkers and other neuropsychological or lesion parameters have not yet been evaluated [17,18], and there were issues of dose optimization and duration of treatment that limit more sophisticated interpretation of the poor results [19,20].

**Fig 4:**
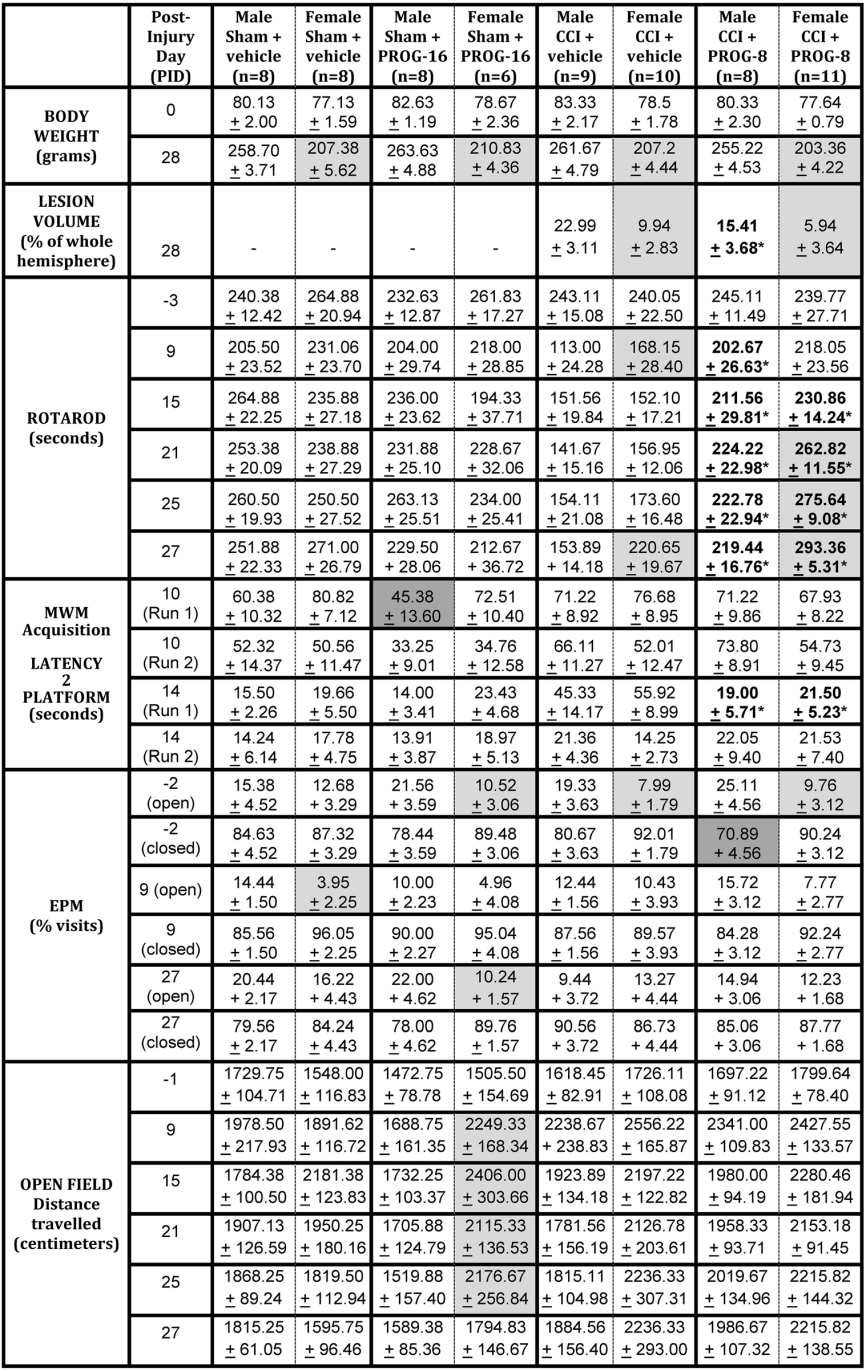
Tabulated comparative deficits between male and female controlled cortical impact injury. Bold numbers with asterisk (*) indicate differences between treatment groups within the same gender, *p* < .05. Darker-shaded cells indicate where male rats either found the hidden MWM platform faster or made fewer visits to the closed arm of the EPM than similarly treated female rat pups (between-gender analysis), *p* < .05. Lighter-shaded cells indicate where female rat pups either weighed less, had smaller lesions, spent less time in the open arm of the EPM, tended to travel longer distances (Open Field), remained on the rotarod longer, or found the MWM platform faster than similarly treated male counterparts (b/w gender analysis), *p* < .05; PID 0 = day of injury but prior to surgery; PID -1 = day(s) before surgery; MWM: Morris Water Maze; CCI: controlled cortical impact; Mean +/- SEM; n = 6–11. Male data (columns 3, 5, 7, and 9) were previously published [26].

The disappointing trial results make it more important to examine the varying efficacy of PROG treatment by sex, age, dosing and duration of treatment as well as to identify the most appropriate dosing and comparable outcome measures [19,20]. Here we compared pre-estrous, prepubescent PND28 female SD rats from the current investigation and PND28 male SD rats from our previous publication [26]. Fig 4 summarizes the comparative analysis of the male and female CCI rat pup data where the specific location of impact and impactor size, velocity, and impactor contact times were identical.

### Sex differences and body weight: Effects of CCI injury and PROG treatment

It has long been known that in peri-adolescent humans, around age 14, a gender-based divergence in mean body weight arises [57]. This difference intensifies and becomes significant during puberty and into young adulthood. In the present study, by PID 28, the sham-injured/vehicle-treated females weighed approximately 50 grams less (Fig 4, row 2, lighter-shaded cells) than male counterparts. These data are not surprising, but it is important to note that whatever sexually dimorphic developmental factors are leading to differences in mean body weight in the maturing human, these differences are preserved in our growing, vehicle-treated control rat pups despite sham surgery. These weight and body size sex differences could affect how a similar dose of a lipophilic hormone like PROG could be metabolized and transported to the brain. These considerations could be especially important in planning for clinical dose optimization with any therapeutic agent and would certainly be important in considering the use of a key sex hormone in developing females. Body weight and mass could very likely affect drug metabolism and other pharmacokinetic factors affecting the response to treatment [19].

Neither CCI injury nor PROG treatment produced any noticeable changes in mean body weight in male or female rat pups, although rats in the Sham+PROG groups weighed slightly more, and CCI+PROG rats weighed slightly less, than their vehicle-treated counterparts (Fig 4, row 1, column 1). Further studies are warranted to determine whether there are long-term differences in mean body weight between sexes as a result of treatment with PROG, or any other neuroprotective drug, for that matter.

### Lesion extent/severity: Effect of PROG treatment

An early study on sex differences and brain injury reported lower post-injury cerebral edema in pseudopregnant female SD rats compared to normally cycling females and males [58–61]. In the present study, lesion reconstruction data indicate significant differences in TBI neuropathology between sexes in the developing rodent. As shown in Fig 4, row 3, compared to males, CCI surgery clearly produced less physical damage to brain tissue (% total hemispheric tissue loss) of juvenile female rats. Previously, in males we observed a significant effect of PROG on mean percent of volumetric tissue loss at all six A/P levels examined, but in females we observed (Fig 1) a significant effect of PROG only at the three anterior levels (+4.5, +3.5, +2.5). Robertson and Saraswatihere [54] were recently surprised to find that their vehicle-treated juvenile SD female CCI rats tended to have smaller lesions compared to male counterparts, but, interestingly, Peterson et al. reported the opposite effect, with males exhibiting far more neuronal sparing after neonatal hypoxic injury [22]. These contradictory data may be the result of the effects of critical periods in development on injury outcome. The inherent differences in response to acquired brain trauma may have a profound effect on treatment effectiveness and delayed functional and morphological sparing [62]. For instance, the PROG treatment that reduced lesion volume by ~33% in SD male rat pups (from 22.99% ± 3.11 to 15.41% ± 3.68) reduced lesion volume by ~40% (from 9.94% ± 2.83 to 5.94% ± 3.64) in age-matched female rats. In this case, owing to the over two-fold difference in the lesion volume compared to age-matched male rats, the injury was too small to see any beneficial treatment effect of PROG on overall lesion volume in the female rats, so the differences between the treated and untreated females was not statistically significant. Given these factors, the potential sex-specific difference in lesion susceptibility (higher in males) and treatment efficacy (lower in females) imply a need for caution when interpreting relative lesion severity and designing injury models, and especially when comparing treatment efficacy across sexes in developing humans.

### Rotarod: Effects of CCI injury and PROG treatment

The rotarod tests motor coordination and vestibular balance [63]. Acute CCI injuries to the unilateral parietal [64] and bilateral mPFC [26] have been independently shown to disrupt rotarod performance in PND17 and PND28 male rats, respectively. In the present study, rotarod performance of sham-injured rat groups did not differ by sex or treatment when tested before 1 month of age (Fig 4, rows 4–9, columns 2–5). In contrast to our findings, Wagner et al. [65] reported that vehicle-treated SD female rats with diffuse axonal injury performed better on the rotarod than vehicle-treated sham-injured males. Here, in contrast to its effect on the sham-injured rats, CCI surgery produced a slightly less sustained deficit on rotarod performance in juvenile females on PID 9 and 27 and PROG treatment was clearly more beneficial in female rats on PID 21, 25, 27 compared to their male counterparts evaluated in our previous study [26] (Fig 4, rows 4–9, columns 6–9). It is possible that exogenous and endogenous PROG levels during puberty synergistically interact to combat a TBI sustained prior to adolescence. Only longer-term tests and gender comparisons from pre- to post-puberty will begin to address this issue.

### Activity level: Effects of CCI injury and PROG treatment

Open Field and EPM tests are used to study hyperactivity and exploratory and anxiety-like behavior rodents [66,67]. Parker and Morinan [68] demonstrated that rodent behavior in the EPM is primarily driven by anxiety-related behaviors in juvenile and adult rats of both sexes. We have previously demonstrated that both EPM and open field results are affected by CCI injury and damage to the mPFC [26,28,31]. In contrast to adult rats, immature rats with moderate brain injury displayed relatively minor changes in activity level compared to controls. We found that female rat pups sporadically spent more time in the open arm of the EPM than males, and PROG-treated sham-injured females tended to be more active than sham-injured males given PROG. Future studies will confirm or discount the strength of these trends.

### Spatial learning: Effects of CCI injury and PROG treatment

The rodent MWM was designed to use spatial learning in rodents as a measure of cognitive, spatial and memory performance [69]. More recently, gender differences have been reported using virtual MWM tasks in young adults (undergraduate students) and prepubescent children [70,71]. While both studies found that males were better at visual tasks, higher levels of anxiety during spatial tasks have been reported in young adult human females [72], and shown to selectively affect hippocampus-dependent learning in children [73]. This spatial anxiety may retard spatial navigation as a function of sex or age, suggesting an increased vulnerability in the developing females.

There were no observed differences among the male and female rats in MWM performance on the first (acquisition) trial (Fig 4 row 10). Similar to the rotarod results, sham surgery and PROG treatment in the intact animals showed no evidence of sex differences in acquiring the task. Others have reported that CCI injury did not affect reversal learning in male or female SD rat pups after excitotoxic lesions of the mPFC [74]. In our study, CCI or PROG treatment had minimal effects on short-term memory, but PROG treatment improved performance on long-term memory tasks by the fifth exposure (on PID 14) equally in female and male CCI rat pups (Fig 4, row 10). This is important because, while the majority of juvenile brain injury studies using hormonal treatment have had encouraging results, longer-term studies have indicated that sustained elevation in neonatal PROG significantly affects sexual behaviors in adulthood in males but not in females [75]. Looking forward, perhaps particularly in females, determining the time when hormonal treatment is appropriate to administer (and during what stage of estrus) following a pediatric TBI could be very important.

### Conclusions

Our data demonstrate that the same injury parameters used to produce a moderate TBI in prepubescent (PND 28) male SD rat pups [26] were sufficient to produce histopathological and functional deficits in female conspecifics. Sex differences were observed in the severity of CCI injury as well as in the effectiveness of post-injury PROG treatment on some, but not all, functional performance outcomes. For neuroprotective strategy/studies we think that it will be important to take into account the TBI severity, sex and hormonal status of the patient at the time treatment is delivered. Preclinical juvenile rodent brain injury data to date suggest that the effects of PROG treatment post-TBI may vary not only by (a) age at time of injury and (b) drug dose/duration, but also by (c) injury type (e.g., diffused, focal, or penetrating brain injury, blast trauma, hypoxia-ischemia, reperfusion injury) and (d) gender [52,56,76–79]. In particular, a given brain injury could have far more deleterious effects in the early stages of life than if the same damage occurs later in development [80,81]. We suggest that if clinical research is to advance, future studies will have to document TBI progression and the effects of hormonal treatment throughout puberty across genders not only on TBI-induced deficits, but also on normal milestones in maturation like puberty onset and gender-specific sexual-social behavior.

## Supporting Information

S1 Fig Dose-response effect of progesterone on weight. Mean body weight (gm) showing weight changes in each group. No significant differences in body weight between groups (*p* > 0.05) were observed. Values are mean ± SEM (n = 6–11 / group). PROG = progesterone.
(TIFF)

S2 Fig Effect of progesterone on elevated plus maze (EPM) activity. Percent of visits to pair of open vs. closed arms. There were no significant differences between groups in the % of visits to the open and closed arms of the EPM maze either pre- or post-surgery (*p’s* > 0.05). * = differences in the arm visited (open vs. closed). Values are mean ± SEM (n = 6–11 / group). PROG = progesterone.
(TIFF)
